# Complete Genome Sequence of *Mycoplasma bovoculi* Strain M165/69^T^ (ATCC 29104)

**DOI:** 10.1128/genomeA.00115-14

**Published:** 2014-02-20

**Authors:** Michael J. Calcutt, Mark F. Foecking

**Affiliations:** Department of Veterinary Pathobiology, University of Missouri, Columbia, Missouri, USA

## Abstract

Bovine ocular infections compromise animal health and result in significant economic losses. *Mycoplasma bovoculi* is an etiological agent of conjunctivitis. Presented here is the 760,240-bp complete genome sequence of the *M. bovoculi* type strain M165/69^T^. An analysis of the deduced proteome provides insights into the adherence and antigenic variation mechanisms of the strain.

## GENOME ANNOUNCEMENT

*Mycoplasma bovoculi* is an etiological agent of bovine conjunctivitis (1) and is also associated with the development of the more serious infection, bovine keratoconjunctivitis (IBK), caused by *Moraxella bovis* (2). Despite its importance, there is a paucity of molecularly characterized features beyond the identification of the adherence related antigen, P94 (3, 4). Presented here is the complete genome sequence of the M165/69^T^ type strain that was isolated in 1968 from a bovine eye in Canada (5, 6).

Total genomic DNA was prepared from *M. bovoculi* M165/69^T^ (obtained from the American Type Culture Collection as ATCC 29104) and subjected to 454 Titanium sequencing at The Genome Institute of Washington University, St. Louis, MO. The resulting reads were assembled *de novo* using Newbler software (Roche), yielding 11 contigs with 156× coverage. An analysis of contig ends together with PCR amplification and amplicon cloning enabled closing of the 760,240-bp genome. After initial automated annotation (PGAP pipeline at the National Center for Biotechnology Information), manual curation was performed, followed by verification of potential pseudogenes by PCR and Sanger sequencing. As a result, the genome comprises 626 genes (579 open reading frames [ORFs] and 12 pseudogenes), including those for 30 tRNAs and one copy for each rRNA (with 5S rRNA gene separated from the 16S-23S rRNA operon). The G+C content is 28.2%.

As anticipated from its 16S rRNA-based phylogeny, most genes in *M. bovoculi* M165/69^T^ exhibit the greatest similarity to those encoded by other sequenced members of the *Mycoplasma neurolyticum* cluster, with those from the caprine pathogen *Mycoplasma conjunctivae* typically being the best match. Although multiple short regions of synteny are present with the latter genome, none are longer than the 27 genes within the *rpsJ-rplQ* locus that predominantly encode ribosomal proteins.

In other sequenced taxa in this cluster, distinctive pairs of ORFs encode posttranslationally processed surface proteins that have been biochemically proven (7, 8) or implicated (9, 10) in mediating adherence to cellular receptors or extracellular matrix components. The *M. bovoculi* genome encodes 7 instances of such gene pairs that are candidate adherence factors for this agent. An array of five genes (*vpbA*, *vpbB*, *vpbC*, *vpbD*, and *vpbE*) encoding paralagous surface proteins (52 to 66% protein identity) represents another potential adaptation feature. Each gene in this novel contingency locus is preceded by a putative promoter containing a homopolymeric thymidine tract that is indicative of phase variation and combinatorial expression patterns (11).

An analysis of the restriction-modification genes identified potentially phase-variable type I and type III systems, each containing a coding sequence harboring poly(GA) repeats that are predicted to undergo stochastic slipped-strand mispairing as reported in other systems (12, 13). A putative HpaI restriction-modification locus is also present, linked to a *repB* pseudogene that likely represents a plasmid integration event. No clustered regularly interspaced short palindromic repeat (CRISPR) units, multicopy insertion sequences, prophages, or integrative conjugative elements were identified.

The genomic sequence is the first for this species and provides a foundation for future investigations of pathoadaptations to the bovine eye. Ultimately, such analyses may allow the development of improved immunological products against conjunctivitis and IBK.

### Nucleotide sequence accession number.

This complete genome sequence has been deposited at DDBJ/EMBL/GenBank under the accession no. CP007154.

## References

[1] Rosenbusch RF Knudtson WU 1980 Bovine mycoplasmal conjunctivitis: experimental reproduction and characterization of the disease. Cornell Vet. 70:307–320 7460567

[2] Postma GC Carfagnini JC Minatel L 2008 *Moraxella bovis* pathogenicity: an update. Comp. Immunol. Microbiol. Infect. Dis. 31:449–458. 10.1016/j.cimid.2008.04.001 18514312

[3] Salih BA Rosenbusch RF 1998 Identification and localization of a 94-kDa membrane protein found in *Mycoplasma bovoculi* strains. Comp. Immunol. Microbiol. Infect. Dis. 21:281–290. 10.1016/S0147-9571(98)00013-7 9775358

[4] Salih BA Rosenbusch RF 1999 Interactions of *Mycoplasma bovoculi* with erythrocytes: role of p94 surface protein. Zentralbl. Veterinarmed. B. 46:323–329 10416366 10.1111/j.1439-0450.1999.tb01237.x

[5] Langford EV Dorward WJ 1969 A mycoplasma isolated from cattle with infectious bovine keratoconjunctivitis. Can. J. Comp. Med. 33:275–279 4243033 PMC1319444

[6] Langford EV Leach RH 1973 Characterization of a mycoplasma isolated from infectious bovine keratoconjunctivitis: *M. bovoculi* sp. nov. Can. J. Microbiol. 19:1435–1444. 10.1139/m73-231 4129282

[7] Bogema DR Deutscher AT Woolley LK Seymour LM Raymond BB Tacchi JL Padula MP Dixon NE Minion FC Jenkins C Walker MJ Djordjevic SP 2012 Characterization of cleavage events in the multifunctional cilium adhesin Mhp684 (P146) reveals a mechanism by which *Mycoplasma hyopneumoniae* regulates surface topography. mBio 3(2):e00282-11. 10.1128/mBio.00282-11 22493032 PMC3322551

[8] Zimmermann L Peterhans E Frey J 2010 RGD motif of lipoprotein T, involved in adhesion of *Mycoplasma conjunctivae* to lamb synovial tissue cells. J. Bacteriol. 192:3773–3779. 10.1128/JB.00253-10 20494988 PMC2897346

[9] Yang F Tang C Wang Y Zhang H Yue H 2011 Genome sequence of *Mycoplasma ovipneumoniae* strain SC01. J. Bacteriol. 193:5018. 10.1128/JB.05363-11 21742877 PMC3165710

[10] Siqueira FM Thompson CE Virginio VG Gonchoroski T Reolon L Almeida LG da Fonsêca MM de Souza R Prosdocimi F Schrank IS Ferreira HB de Vasconcelos AT Zaha A 2013 New insights on the biology of swine respiratory tract mycoplasmas from a comparative genome analysis. BMC Genomics 14:175. 10.1186/1471-2164-14-175 23497205 PMC3610235

[11] Yogev D Rosengarten R Watson-McKown R Wise KS 1991 Molecular basis of *Mycoplasma* surface antigenic variation: a novel set of divergent genes undergo spontaneous mutation of periodic coding regions and 5′ regulatory sequences. EMBO J. 10:4069–4079 1721868 10.1002/j.1460-2075.1991.tb04983.x PMC453155

[12] Srikhanta YN Maguire TL Stacey KJ Grimmond SM Jennings MP 2005 The phasevarion: a genetic system controlling coordinated, random switching of expression of multiple genes. Proc. Natl. Acad. Sci. U. S. A. 102:5547–5551. 10.1073/pnas.0501169102 15802471 PMC556257

[13] Srikhanta YN Gorrell RJ Steen JA Gawthorne JA Kwok T Grimmond SM Robins-Browne RM Jennings MP 2011 Phasevarion mediated epigenetic gene regulation in *Helicobacter pylori*. PLoS One 6:e27569. 10.1371/journal.pone.0027569 22162751 PMC3230613

